# Recurrent neonatal seizures increase tonic inhibition and respond to enhancers of **δ**-containing GABA_A_ receptors

**DOI:** 10.1172/jci.insight.196152

**Published:** 2025-09-16

**Authors:** Gage T. Liddiard, Gordon F. Buchanan, Mark L. Schultz, Joseph Glykys

**Affiliations:** 1 Stead Family Department of Pediatrics,; 2 Interdisciplinary Graduate Program in Neuroscience,; 3 Department of Neurology,; 4 Medical Scientist Training Program, and; 5 Iowa Neuroscience Institute, The University of Iowa, Iowa City, Iowa, USA.

**Keywords:** Cell biology, Neuroscience, Epilepsy, Ion channels, Seizures

## Abstract

About one-third of neonatal seizures do not respond to the first-line anticonvulsant phenobarbital, which activates phasic inhibition and whose effectiveness decreases over time. Whether enhancing tonic inhibition can treat refractory seizures or status epilepticus in neonates remains uncertain. We evaluated the effect of recurrent seizure-like events (SLE) on α5– and δ–GABA_A_ receptor (α5- and δ-GABA_A_R) subunit expression and tonic inhibition in neonatal C57BL/6J mice (P6–9, both sexes) using acute brain slices. We investigated the impact of THIP (gaboxadol) on neonatal behavioral seizures, neuronal apoptosis, and neurodegeneration in vivo. We found neonatal neocortical expression of α5- and δ-GABA_A_R subunits. Blocking α5-GABA_A_Rs with L-655,708 did not affect acute neonatal SLE, whereas enhancing δ-GABA_A_Rs with THDOC, a neurosteroid, reduced them. The α5- and δ-GABA_A_R membrane expression increased after 8 hours of neonatal SLE and correlated with increased δ-mediated conductance but not α5-mediated conductance. Enhancing tonic inhibition was more effective in reducing recurrent neonatal SLE (8 hours) compared with early treatment. Increasing tonic inhibition reduced the duration, severity, and number of kainic acid–induced in vivo neonatal behavioral seizures without increasing neurodegeneration or apoptosis. We conclude that recurrent neonatal seizures increase tonic inhibition. Therefore, enhancing tonic inhibition may be a treatment strategy for prolonged neonatal status epilepticus.

## Abstract 2

Neonatal status epilepticus increases tonic inhibition mediated by extrasynaptic GABAA receptors and responds to δ subunit selective GABAergic enhancers

## Graphical abstract

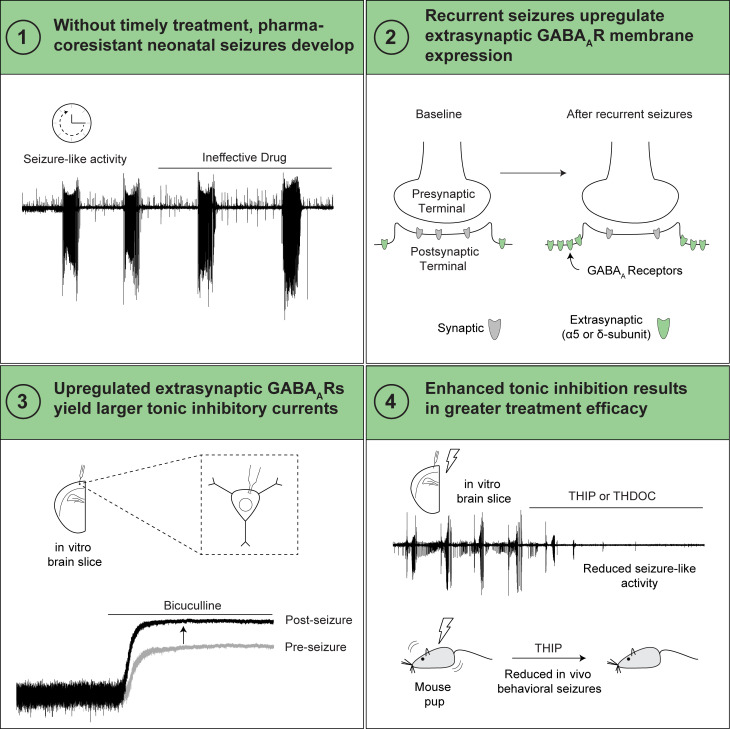

## Introduction

Approximately 30%–50% of neonatal seizures resist treatment (1, 2). Early recognition and treatment are crucial, as seizures become harder to manage over time. Yet, their detection and treatment are not always immediate, especially in medically underserved areas (3). Despite advancements in the development of new antiseizure medications, there has not been a significant change in first-line neonatal seizure treatment for nearly 50 years (4). Phenobarbital is still the first-line treatment for neonatal seizures, even though it fails in 30%–50% of cases, especially when diagnosis and intervention are delayed (1, 4–6). This poor effect of phenobarbital is also observed in rodents ex vivo and in vivo (7–9). Therefore, there is a need for new therapies, especially in patients in which treatment is delayed and pharmacoresistance develops.

Gamma-aminobutyric acid (GABA) is the brain’s primary inhibitory neurotransmitter. However, its actions depend on the relationship between the GABA reversal potential (E_GABA_), which is mainly determined by the intraneuronal chloride concentration ([Cl^–^]_i_) (10), and the neuron’s resting membrane potential. Neonatal mice have elevated [Cl^–^]_i_ compared with adults, resulting in depolarizing GABA actions (11, 12). Moreover, neonatal seizures induce activity-dependent Cl^–^ accumulation, further increasing [Cl^–^]_i_ and the depolarizing actions of GABA (8, 13).

GABA_A_ receptors (GABA_A_Rs) are pentameric assemblies situated in the synaptic and extrasynaptic neuronal compartments. Synaptic GABA_A_Rs mediate phasic conductance without exerting a lasting influence on the resting membrane potential (14, 15). In contrast, extrasynaptic GABA_A_Rs mediate tonic conductances and have a lasting impact on the resting membrane potential and the neuronal gain (14, 16, 17). The extrasynaptic GABA_A_Rs have a unique subunit composition. The δ subunit–containing GABA_A_Rs are exclusively extrasynaptic, while the α5 subunit–containing GABA_A_Rs are primarily extrasynaptic (18–20). The expression of these subunits is detectable postbirth in the hippocampus and neocortex (21, 22), which are common areas for seizure initiation and propagation (23).

Extrasynaptic GABA_A_R modulation may be one solution for the pharmacoresistance observed after prolonged status epilepticus. During status epilepticus, synaptic GABA_A_Rs are rapidly internalized, hindering the efficacy of traditional GABAergic agents like phenobarbital and diazepam (24–27). Meanwhile, concomitant with the internalization of synaptic GABA_A_Rs, the expression of extrasynaptic GABA_A_Rs either remains constant or increases (27–30). However, it remains unclear whether similar alterations in extrasynaptic GABA_A_R expression, leading to functional changes in tonic inhibition, occur with recurrent seizures (prolonged status epilepticus) in the neonatal brain.

In neonatal mice, we previously showed that CA1 and neocortical pyramidal cells have a tonic inhibitory current. This current, when enhanced by the δ subunit–selective agonist THIP [4,5,6,7-tetrahydroisoxazolo(5,4-c)pyridin-3-ol, gaboxadol], also known as gaboxadol, reduced neonatal seizure-like events (SLE) in acute brain slices (31). However, it remains unclear whether increasing tonic inhibition will still benefit recurrent SLE persisting for hours, akin to clinical status epilepticus.

In this study, we assessed the alterations in the α5 and δ subunit membrane expression, the tonic conductances they mediate, and the impact of enhanced tonic inhibition following recurrent neonatal SLE in acute brain slices. We also evaluated the effect of THIP on behavioral seizures in vivo. Our findings revealed that enhancing tonic inhibition reduced recurrent neonatal SLE, even when it persisted for 8 hours. Moreover, we demonstrated that enhancing tonic inhibition significantly reduced kainic acid–induced (KA-induced) neonatal behavioral seizures and did not worsen neuronal degeneration or apoptosis in vivo. Therefore, augmenting tonic inhibition may be a viable treatment for neonatal status epilepticus, without losing its efficacy over time.

## Results

### Expression of the α5 and δ GABA_A_R subunits in the neonatal mouse neocortex.

**Figure 1:**
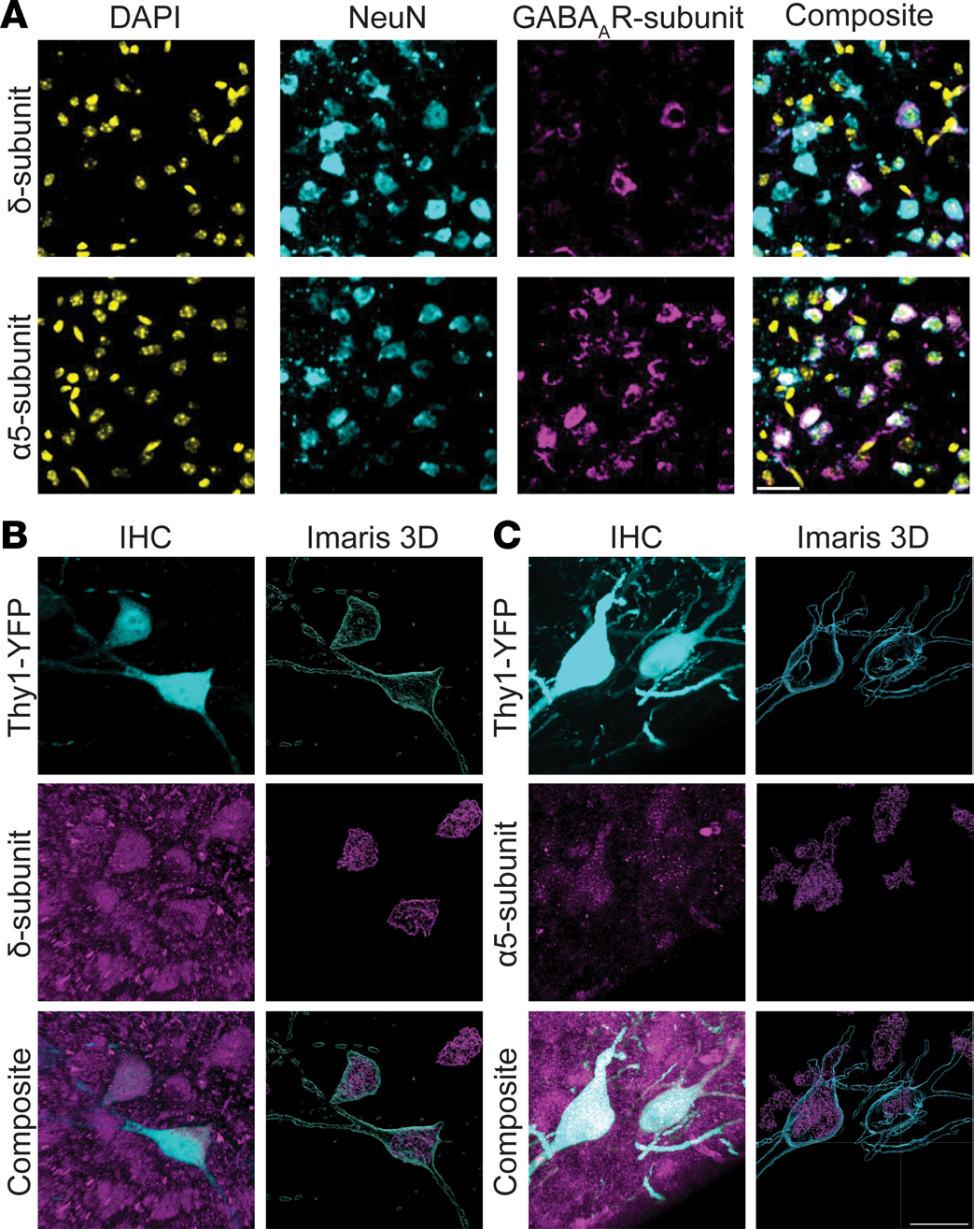
The α5- and δ-GABA_A_R subunits are expressed in the neonatal neocortex. (**A**) Representative pseudocolored images of the δ subunit (top) and α5 subunit (bottom) in the neonatal neocortex. Columns: DAPI (yellow), NeuN (cyan), subunit (magenta), and composite. P8 mice. (**B** and **C**) Representative pseudocolored images of somatic neuronal δ (**B**) and α5 subunit (**C**) expression. *Left:* Z-stack (30 μm slice, 2 μm steps) from each group. *Right*: Imaris 3D reconstruction highlighting shape and overlap. *Rows:* YFP-expressing neurons (cyan), subunit (magenta), and composite. P12. Scale bars, 25 μm. YFP, yellow fluorescence protein.

The mRNA expression of the α5 and δ GABA_A_R subunits is detectable postbirth in the hippocampus and neocortex (21, 22). We used immunofluorescence to confirm the neuronal expression of the α5 and δ subunits in acute brain slices from neonatal (P6–8) C57BL/6J mouse neocortex (Figure 1). We observed that both the α5 and δ subunits displayed neuronal expression in the neonatal neocortex (Figure 1A). This neuronal localization was confirmed by visualizing 3-dimensional reconstructions of the α5 and δ subunits overlaid on YFP-expressing neocortical neurons (Thy1-YFP mouse line, P12) (Figure 1, B and C).

### Blocking the α5-containing GABA_A_Rs with L-655,708 does not worsen neonatal SLE.

**Figure 2:**
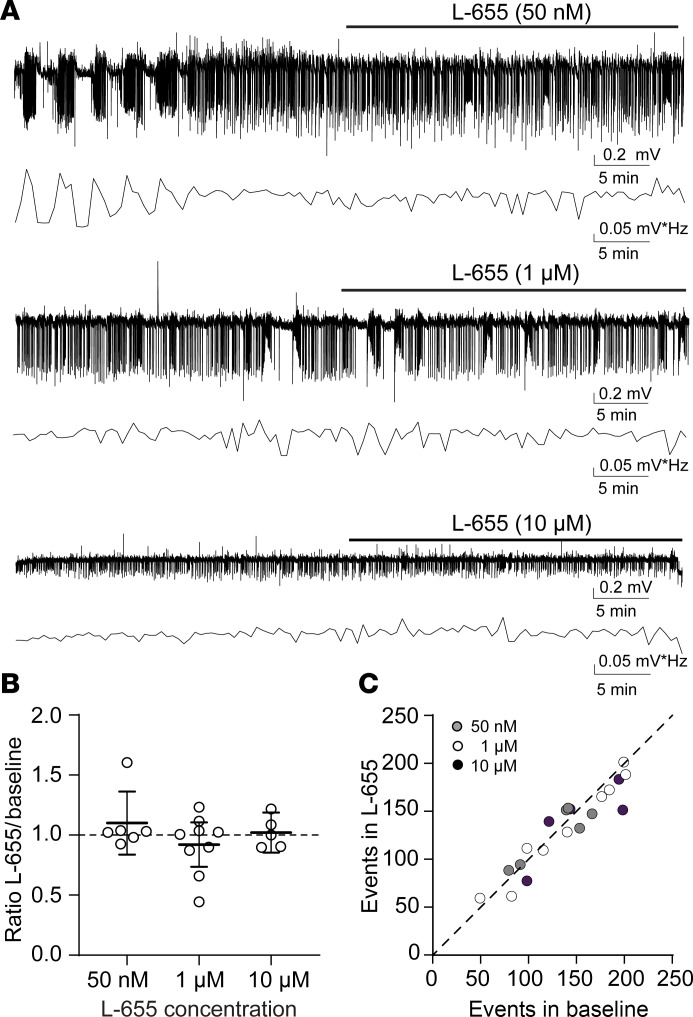
Inhibiting the α5-containing GABA_A_R does not worsen seizure-like events in the neonatal neocortex. (**A**) Seizure-like events induced by Low-Mg^2+^ artificial CSF (aCSF) recorded in the neocortical layer IV/V of acute brain slices (P8) using an extracellular field electrode before and after L-655 at 50 nM, 1 μM, and 10 μM. The corresponding Fast Fourier Transform (FFT) power area calculated every 30 seconds is displayed below the traces. (**B**) SLE FFT power ratios (L-655/baseline) in the neonatal neocortex using different L-655 concentrations. One-way ANOVA, *F*(2, 17) = 1.185, *P* = 0.33, *n* = 6, 9, 5 recordings, respectively. (**C**) Number of SLE events during L-655 (*y* axis) versus baseline (*x* axis) under different L-655 concentrations. Circles: individual slices. Mean ± 95% CI. The line represents a slope of 1.

Our previous findings showed that enhancing tonic inhibition with the δ subunit–selective agonist THIP reduces neonatal SLE in acute brain slices (31). However, the neonatal mouse brain also expresses the α5 subunit (Figure 1). Therefore, we hypothesized that applying the α5-specific inverse agonist L-655,708 (L-655) (32) would worsen neonatal SLE. We recorded SLE in acute brain slices from C57BL/6J mice (neocortex layers IV/V, P6–8) using the Low-Mg^2+^ model to induce epileptiform activity (33–35) and quantified their power using a Fast Fourier Transform (FFT) as previously done (7, 31). After establishing a stable 30-minute baseline, we perfused different concentrations of L-655 (50 nM, 1 μM, and 10 μM; Figure 2A). Regardless of the concentration applied, we did not observe a significant change in SLE power (Figure 2B). Similarly, we did not observe any significant change in the number of SLE events (*P* = 0.92, 0.18, 0.41, respectively, paired *t* test, Figure 2C) or SLE amplitudes (*P* = 0.88, 0.160 paired *t* test; >0.999 Wilcoxon test, respectively, *n* = 6, 9, 5) with different L-655 concentrations compared with baseline. Therefore, although the α5-GABA_A_R subunit is expressed in the neonatal neocortex, blocking it did not exacerbate SLE in this brain region.

### Enhancing tonic inhibition with THDOC reduces SLE in the neonatal neocortex.

**Figure 3:**
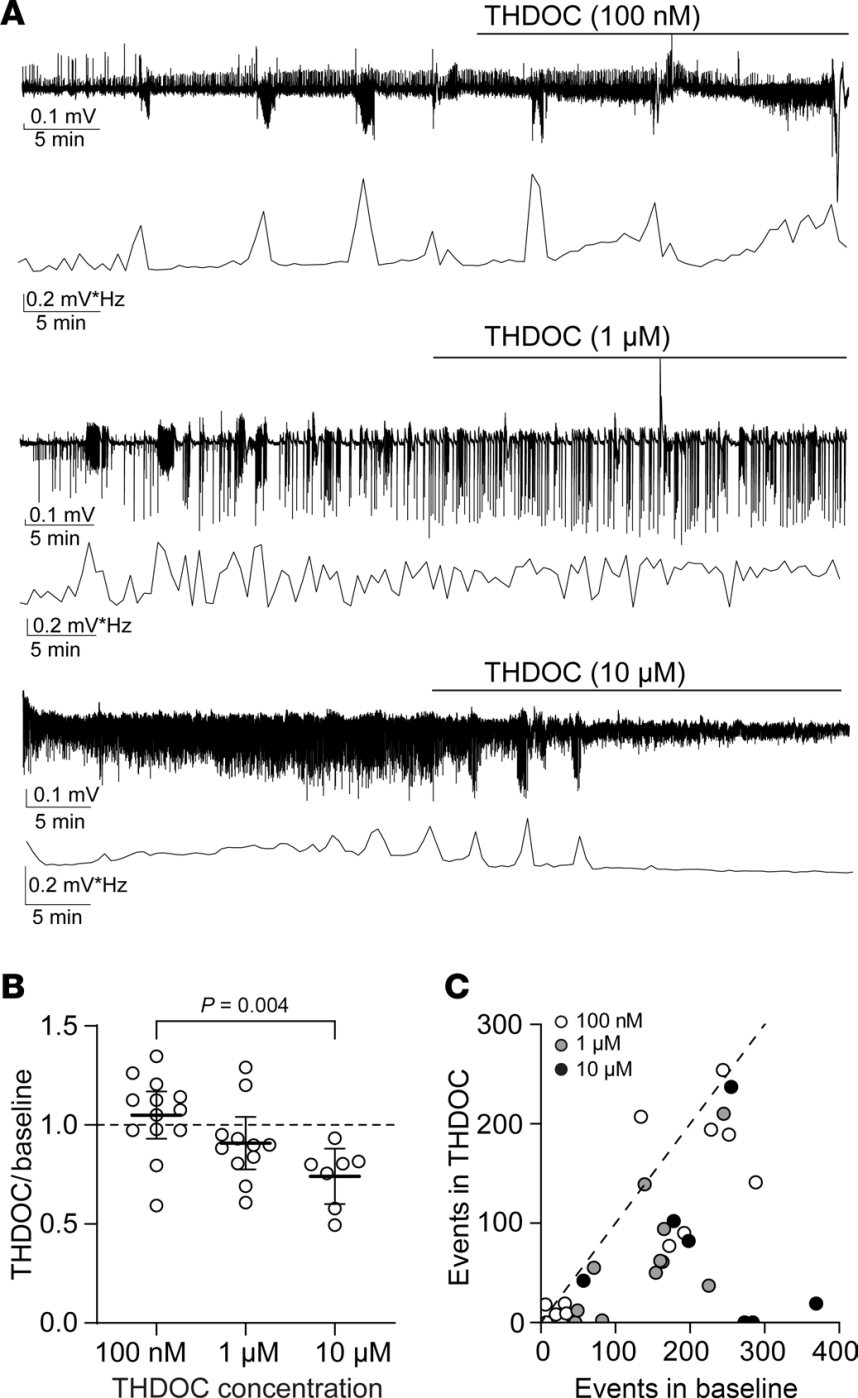
Enhancing tonic inhibition with the neurosteroid analog THDOC reduces neonatal seizure-like events. (**A**) Seizure-like events induced by Low-Mg^2+^ aCSF in the neocortex layer IV/V recorded using an extracellular field electrode in acute brain slices (P8), with the corresponding FFT power area calculated every 30 seconds for THDOC 100 nM, 1 μM, and 10 μM. (**B**) FFT power ratios in the neonatal neocortex using different THDOC concentrations. One-way ANOVA, *F*(2,28) = 6.253, *P* = 0.006, Tukey’s post hoc test is shown in the graph; *n* = 13, 11, 7, respectively. (**C**) Number of SLE during THDOC (*y* axis) versus baseline (*x* axis). Circles: individual slices. Mean ± 95% CI. The line represents a slope of 1.

We next evaluated whether the THIP-mediated SLE reduction we observed previously (31) is drug specific or a property of augmenting δ-GABA_A_R–mediated tonic inhibition. We studied the actions of the neurosteroid 3α,21-Dihydroxy-5α-pregnan-20-one (THDOC), another δ-GABA_A_R–selective agonist (36). Using the Low-Mg^2+^ model to induce neocortical SLE in acute brain slices (P6–8), we determined the effect of different THDOC concentrations on SLE (Figure 3A). We did not observe a change in neonatal SLE power at the δ-GABA_A_R–specific concentrations of 100 nM and 1 μM. In contrast, a significant reduction in FFT power of 26% (95% CI 12–40) was noted with 10 μM (Figure 3B). THDOC reduced the number of SLE at 100 nM (*P* = 0.034, Wilcoxon test, *n* = 13), 1 μM (*P* = 0.003, paired *t* test, *n* = 11), and 10 μM (*P* = 0.040, paired *t* test, *n* = 7; Figure 3C) without altering SLE amplitude at any concentration (*P* = 0.497, 0.426 Wilcoxon test; 0.396, paired *t* test, respectively; *n* = 13, 11, 7). Thus, similar to our prior finding with THIP (31), enhancing tonic inhibition with THDOC also led to a reduction in SLE during the neonatal age.

### The membrane expression of the α5- and δ-GABA_A_R subunits increases after 8 hours of recurrent neonatal SLE.

**Figure 4:**
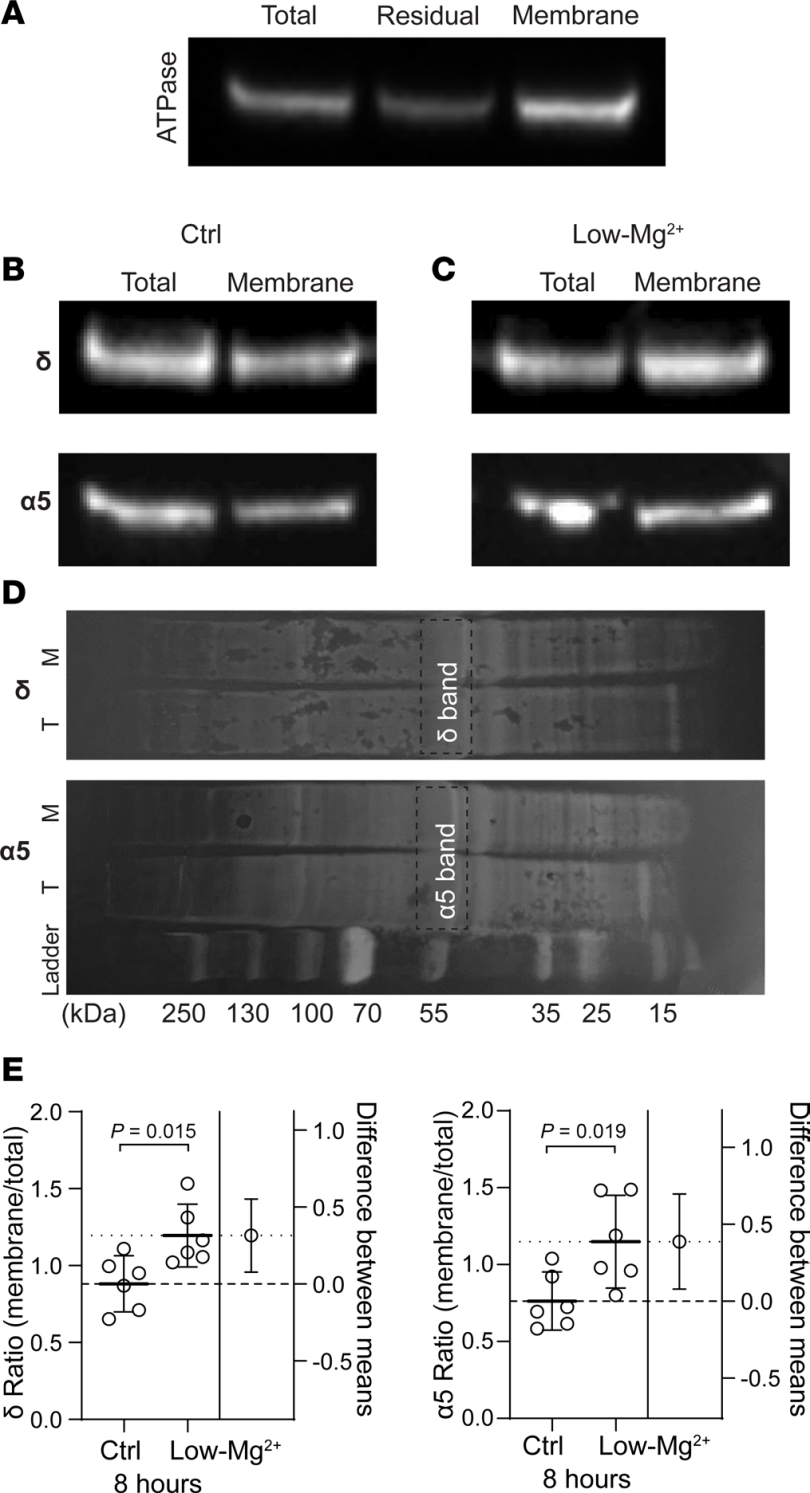
Recurrent neonatal seizure-like events for 8 hours increase the membrane expression of α5- and δ-GABA_A_R subunits. (**A**) Western blot showing enrichment of the membrane-bound Na^+^/K^+^ ATPase in the membrane column using the fractionation kit. (**B**) Sample Western blots of the α5- and δ-GABA_A_R subunits from acute brain slices incubated in aCSF (Ctrl). Same lysate as **A**. (**C**) Sample as in **B** but from acute brain slices incubated in Low-Mg^2+^ aCSF. (**D**) Example amido black total protein stain of δ and α5 subunit lanes showing uniform loading in aCSF (T, total; M, membrane). Expected MW for each subunit is around 55 kDa (dashed boxes). (**E**) *Left*, ratio of the membrane to total δ subunit in aCSF- and Low-Mg^2+^–treated slices. aCSF: 0.88 [0.70, 1.06], Low-Mg^2+^: 1.20 [0.99, 1.40], unpaired *t* test, *n* = 6 animals, 6 blots. *Right*, ratio of membrane to total α5 subunit in aCSF- and Low-Mg^2+^–treated slices. aCSF: 0.76 [0.57, 0.95], Low-Mg^2+^: 1.15 [0.85, 1.45], unpaired *t* test, *n* = 6 mice, 6 blots. Circles: lysate ratios. Lines: mean ± 95% CI.

In adult mice, extrasynaptic GABA_A_R expression is maintained or increased during models of status epilepticus (27–30). However, it is unclear whether similar changes occur after status epilepticus in neonatal mice. Therefore, we assessed whether 8 hours of recurrent SLE (similar to prolonged status epilepticus) in the neonatal neocortex (P6–8) changes the expression of the α5- and δ-GABA_A_R subunits. Acute brain slices were incubated for 8 hours in regular aCSF (Ctrl) or Low-Mg^2+^ aCSF (SLE). Afterward, we fractionated the neocortex from each group to isolate the membrane component (Figure 4A) and quantified the expression of α5- and δ-GABA_A_R subunits between this compartment and the total expression (Figure 4, B–D). Compared with an incubation in aCSF for 8 hours, the α5 and the δ subunits increased their membrane-to-total expression ratio after 8 hours of SLE (Figure 4E). As total protein concentration did not change (α5: *P* = 0.145, δ: *P* = 0.981, 1-sample *t* test), this is likely a result of increased trafficking of already available subunits into the membrane rather than increased protein synthesis. Thus, recurrent SLE for 8 hours, akin to status epilepticus, increased the expression of the α5- and δ-GABA_A_R subunits in the neonatal neocortex.

### Tonic conductances increase after 8 hours of recurrent neonatal SLE.

**Figure 5:**
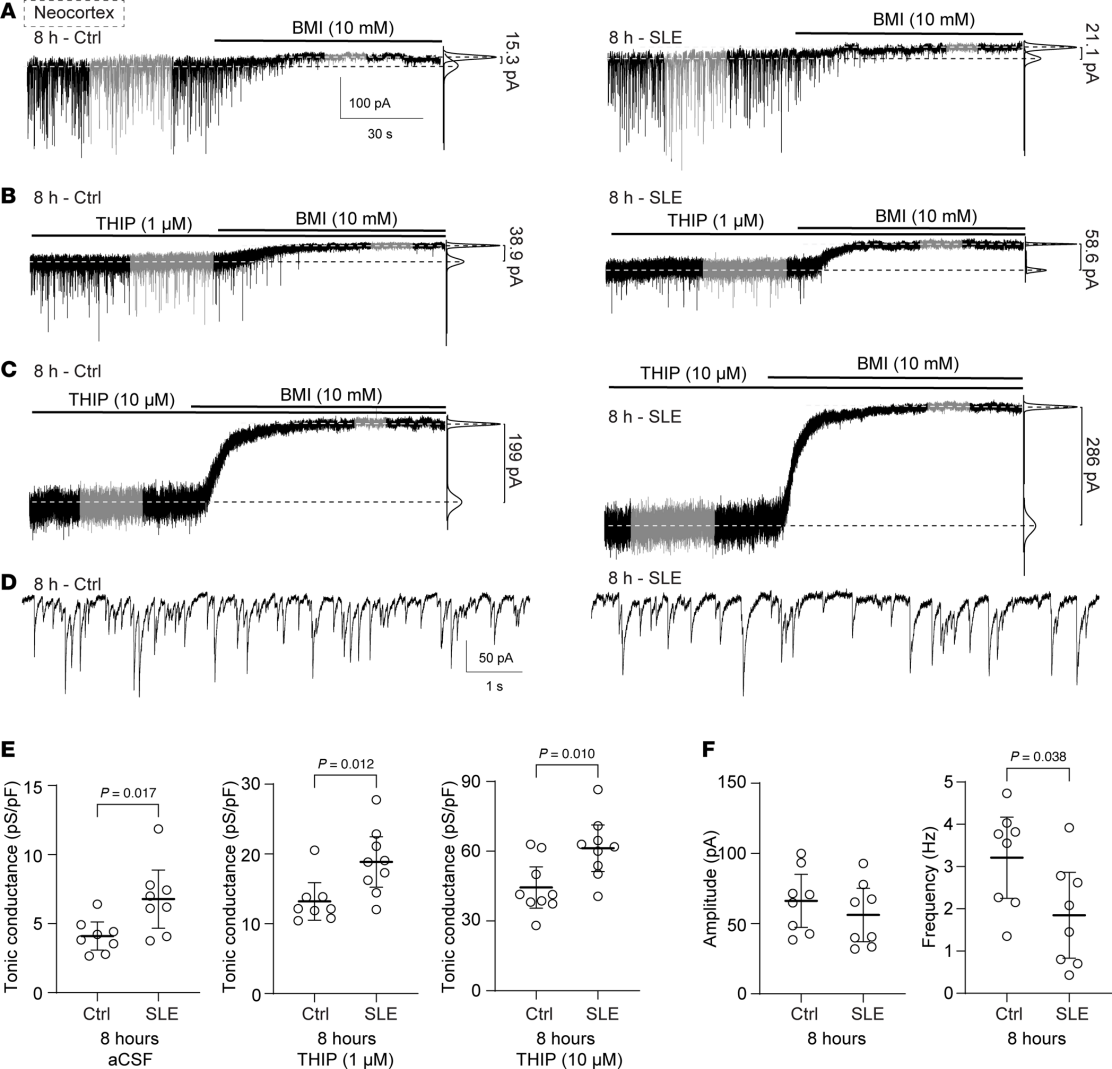
The total and δ-GABA_A_R–mediated tonic conductance increases in the neocortex after 8 hours of recurrent neonatal seizure-like events. Representative whole-cell voltage-clamp recordings of a neocortical pyramidal cell (layer V) without prior seizure incubation (Ctrl, *left*) and after 8 hours of SLE (Low-Mg^2+^, *right*) during perfusion of aCSF (**A**), THIP 1 μM (**B**), and THIP 10 μM (**C**), V_h_ –70 mV. Top lines indicate drug perfusions; right lines indicate fits to the all-points histogram under each condition. (**D**) Example of spontaneous inhibitory postsynaptic currents in aCSF during control and SLE conditions at 8 hours. (**E**) Tonic inhibitory conductances in the neocortex under aCSF (Ctrl: 4.10 pS/pF [3.08, 5.11], SLE: 6.78 pS/pF [4.68, 8.88], unpaired *t* test, *n* = 8, 8), THIP 1 μM (Ctrl: 13.2 pS/pF [10.5, 15.9], SLE: 18.9 pS/pF [15.3, 22.5], unpaired *t* test, *n* = 8, 9), and THIP 10 μM (Ctrl: 44.4 pS/pF [35.5, 53.3], SLE: 61.3 pS/pF [51.3, 71.3], unpaired *t* test, *n* = 9, 9). Note the increase in tonic current with THIP (different *y* axis). (**F**) Spontaneous inhibitory postsynaptic current amplitude (Ctrl: 66.2 pA [47.4, 85.1], SLE: 57.9 pA [35.9, 80.0], *P* = 0.51, unpaired *t* test) and frequency (Ctrl: 3.21 Hz [2.25, 4.17], SLE: 1.85 Hz [0.83, 2.87], unpaired *t* test), *n* = 8, both groups. *Not pictured*: Rise time (Mann-Whitney test, *P* = 0.44) and decay (unpaired *t* test, *P* = 0.58). White circles: individual slices. Black lines: mean ± 95% CI.

We next evaluated if the increase in α5- and δ-GABA_A_R subunit membrane receptor expression correlates with enhanced tonic currents using whole-cell patch-clamp current recordings (Figure 5). Again, we incubated acute neonatal brain slices for 8 hours in aCSF (Ctrl) or Low-Mg^2+^ aCSF (Figure 5, A–C, left and right, respectively). We then measured tonic inhibitory currents in neocortical (layers IV/V) pyramidal neurons in regular aCSF (Figure 5A), under THIP at 1 μM to enhance δ-mediated currents (Figure 5B), or under THIP at 10 μM (Figure 5C), akin to our effective dose that reduces acute-onset SLE (31). Recordings were conducted in aCSF lacking GABA and containing 2 mM kynurenic acid to block ionotropic glutamate receptors. Again, we observed an increase in tonic inhibitory conductances with THIP, as we previously showed (31). Notably, after 8 hours of SLE, tonic conductances were significantly increased in all groups compared with the nonseizing aCSF control group (Figure 5E). The spontaneous inhibitory postsynaptic currents between control and 8 hours of SLE (no THIP, Figure 5D) displayed no significant change in rise time, decay, or amplitude but a significant decrease in spontaneous inhibitory postsynaptic current frequency (Figure 5F). Thus, the increased membrane expression of extrasynaptic GABA_A_R subunits after 8 hours of recurrent SLE (Figure 4E) had a functional correlation to an increase in tonic inhibitory conductance, which responded to THIP.

**Figure 6:**
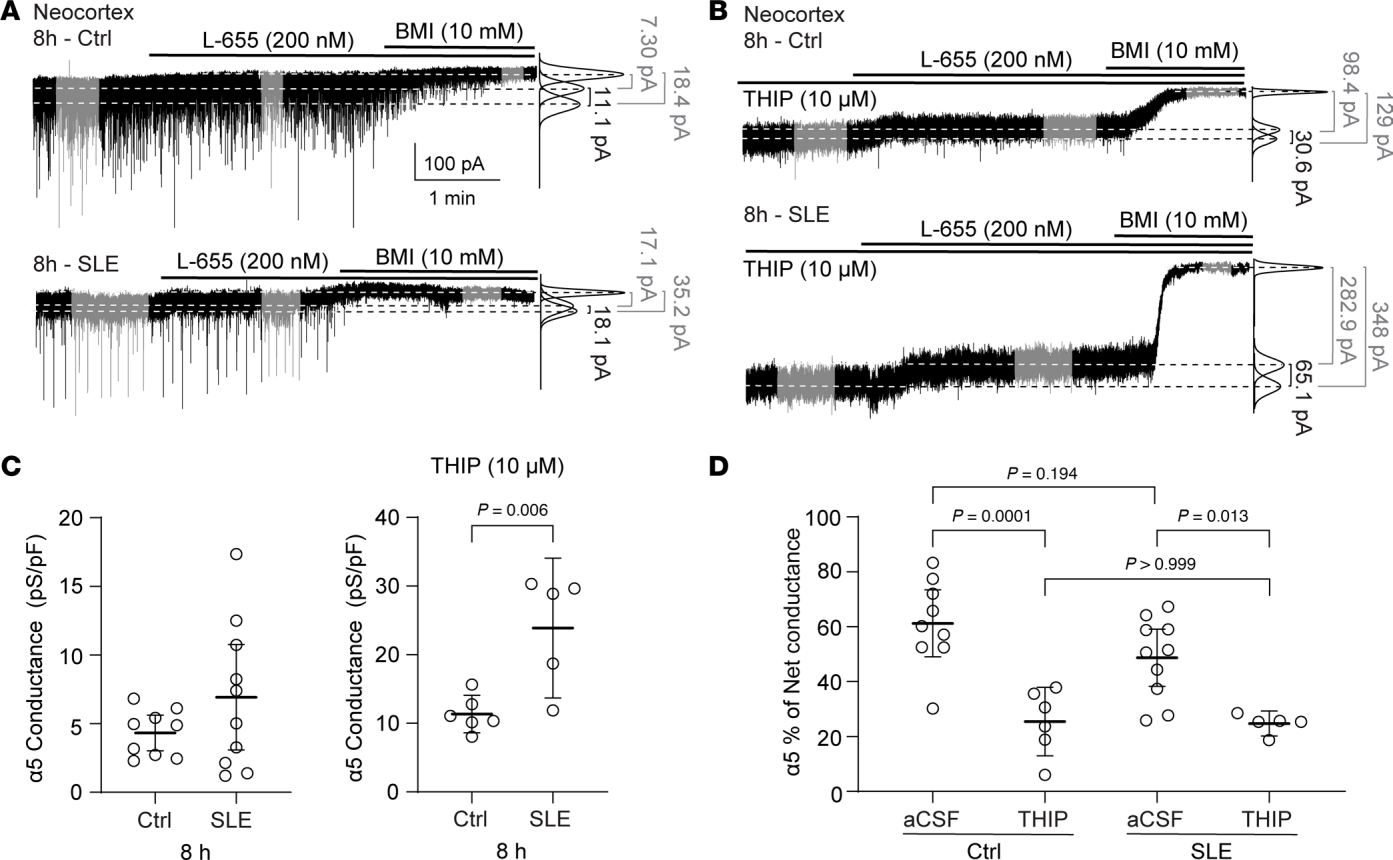
The α5-GABA_A_R–mediated currents increase after recurrent neonatal seizure-like events, but their proportion remains the same. Representative whole-cell voltage-clamp recordings of a neocortical pyramidal cell (layer V) without seizure incubation (Ctrl, *top*) and after 8 hours of Low-Mg^2+^ (SLE, *below*), during perfusion of aCSF (**A**) or THIP 10 μM (**B**) before the application of L-655 (200 nM), V_h_ –70 mV. Top line indicates various drug perfusions; right lines indicate fits to the all-points histograms under each condition (black = α5-mediated current blocked by L-655, gray = net and residual currents). (**C**) α5-mediated tonic conductances with no THIP (Ctrl: 4.32 pS/pF [3.02, 5.63], SLE: 6.93 pS/pF [3.09, 10.8], *P* = 0.182, unpaired *t* test, *n* = 9, 10) and α5-mediated tonic conductances under 10 μM THIP (Ctrl: 11.3 pS/pF [8.61, 14.1], SLE: 23.9 pS/pF [13.7, 34.1], unpaired *t* test, *n* = 6, 5). (**D**) Percent conductance mediated by α5-containing GABA_A_Rs with and without THIP (control: 61.2% [49, 73.5] and 25.5% [13.0, 37.9], SLE: 48.7% [38.3, 59.1] and 24.8% [20.2, 29.3], 1-way ANOVA, *F*(3, 26) = 12, *P* < 0.0001; Šídák’s multiple comparisons test; *n* = 9, 6, 10, 5, respectively). Circles: individual recordings. Black lines: mean ± 95% CI.

Following the increase in overall and δ-mediated tonic conductance after recurrent SLE, we assessed the role of α5-containing GABA_A_R in the tonic conductances. Using whole-cell patch-clamp and the same incubation paradigm, we measured the α5 component of the tonic inhibitory conductances by blocking it with L-655 in regular aCSF and under THIP 10 μM (Figure 6, A and B, respectively). After 8 hours of SLE, neither the α5-mediated tonic conductance (revealed by L-655) nor its proportion to total conductance changed between SLE and control (Figure 6, C and D), suggesting that while the expression of this subunit increased, its functional proportion to total tonic current did not change. Under THIP 10 μM, the α5-mediated tonic conductance also increased during SLE (Figure 6C, right). However, its proportion to total tonic conductance was smaller in THIP in both control and SLE (Figure 6D). These observations suggest that while α5-mediated tonic conductance is present, its proportion to total tonic conductance does not increase with recurrent SLE, and that THIP at 10 μM preferentially increases δ-containing GABA_A_Rs.

### Enhancing tonic inhibition reduces SLE to a greater degree during recurrent neonatal SLE.

**Figure 7:**
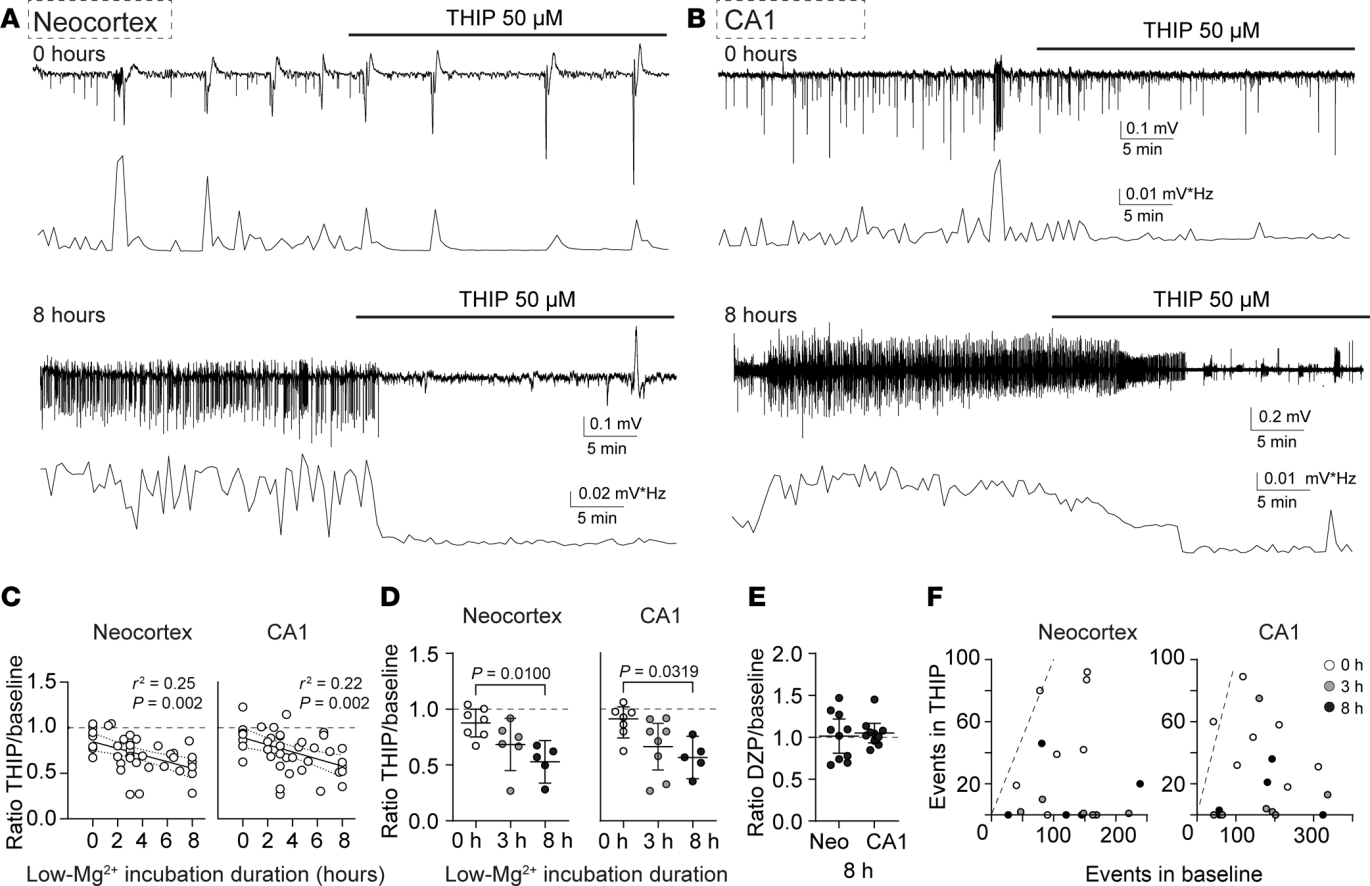
Enhancing tonic inhibition is more effective after recurrent neonatal seizure-like events. *Top:* Seizure-like events induced by Low-Mg^2+^ aCSF in the neocortex, layer IV/V (**A**), and CA1 stratum pyramidale (**B**) recorded with an extracellular field electrode in acute brain slices (P8). *Below*: FFT power area from top traces calculated every 30 seconds. The upper group was recorded with no prior Low-Mg^2+^ incubation, while the lower group was after 8 hours of incubation. (**C**) FFT power ratios versus seizure incubation duration in the neonatal neocortex and CA1 region. Linear regression, *n* = 37 neocortices (slope = –0.037), *n* = 41 CA1 regions (slope = –0.039). Dashed lines ± 95% CI. (**D**) THIP’s effect between slices at 0, 3, and after 8 hours in Low-Mg^2+^. Neocortex: 0 hours: 0.88 [0.75, 1.0], 3 hours: 0.68 [0.45, 0.92], 8 hours: 0.53 [0.34, 0.72]; 1-way ANOVA, *F*(2, 15) = 6.009, *P* = 0.012; *n* = 7, 6, 5 respectively. CA1 region: 0 hours: 0.91 [0.74, 1.08], 3 hours: 0.66 [0.45, 0.87], 8 hours: 0.57 [0.38, 0.76]; 1-way ANOVA, *F*(2, 17) = 4.604, *P* = 0.025, *n* = 7, 8, 5 respectively. (**E**) Diazepam’s (DZP) effect on slices after 8 hours in Low-Mg^2+^. Neocortex: 1.01 [0.98, 1.33], *P* = 0.88, 1-sample *t* test, *n* = 10; CA1 region: 1.05 [0.93, 1.17], *P* = 0.35, 1-sample *t* test, *n* = 10. (**F**) Number of SLE during THIP (*y* axis) versus baseline (*x* axis) in the pup neocortex and CA1 region at 0, 3, and 8 hours of Low-Mg^2+^. The line represents a slope of 1. Circles: individual slices. Black lines: mean ± 95% CI.

After observing that tonic inhibition increased after 8 hours of recurrent SLE in the neonatal neocortex, we hypothesized that enhancing tonic inhibition would be more effective at decreasing SLE at later time points. To evaluate this hypothesis, we recorded SLE using extracellular field electrodes in acute neonatal brain slices (P6–8) containing the neocortex (Figure 7A) and hippocampal CA1 area (Figure 7B) and incubated them in Low-Mg^2+^ aCSF between 0 and 8 hours. After a 30-minute stable SLE baseline, we perfused THIP (50 μM), a dose effective in decreasing acute SLE (31). Our recordings showed greater reductions in SLE power by THIP in slices exposed to Low-Mg^2+^ for longer incubation times (Figure 7C). When comparing 3 time points — no prior incubation versus 3 or 8 hours of Low-Mg^2+^ aCSF — THIP had a larger effect in the 8-hour group in both the neocortex and CA1 area (Figure 7D). In contrast, at the 8-hour time point, diazepam (2 μM), a benzodiazepine that acts on synaptic GABA_A_Rs, was completely ineffective in both brain regions (Figure 7E). There was also a decrease in the number of SLE in both regions with THIP at 0, 3, and 8 hours (Figure 7F; neocortex: *P* = 0.006 paired *t* test, *P* = 0.031 Wilcoxon test, *P* = 0.038 paired *t* test, *n* = 7, 6, 5 respectively; CA1: *P* = 0.025 paired *t* test, *P* = 0.008 Wilcoxon test, *P* = 0.037 paired *t* test, *n* = 7, 8, 5 respectively) but no change in SLE amplitude (neocortex: *P* = 0.469, 0.688, 0.063, Wilcoxon test, *n* = 7, 6, 5 respectively; CA1: *P* = 0.578, 0.195, 0.438, Wilcoxon test, *n* = 7, 8, 5 respectively). Therefore, our results suggest that enhancing tonic inhibition is more effective in treating prolonged and repetitive SLE in neonates than at earlier time points.

### Increasing tonic inhibition decreases neonatal behavioral seizures in vivo with no surge in neurodegeneration or apoptosis.

**Figure 8:**
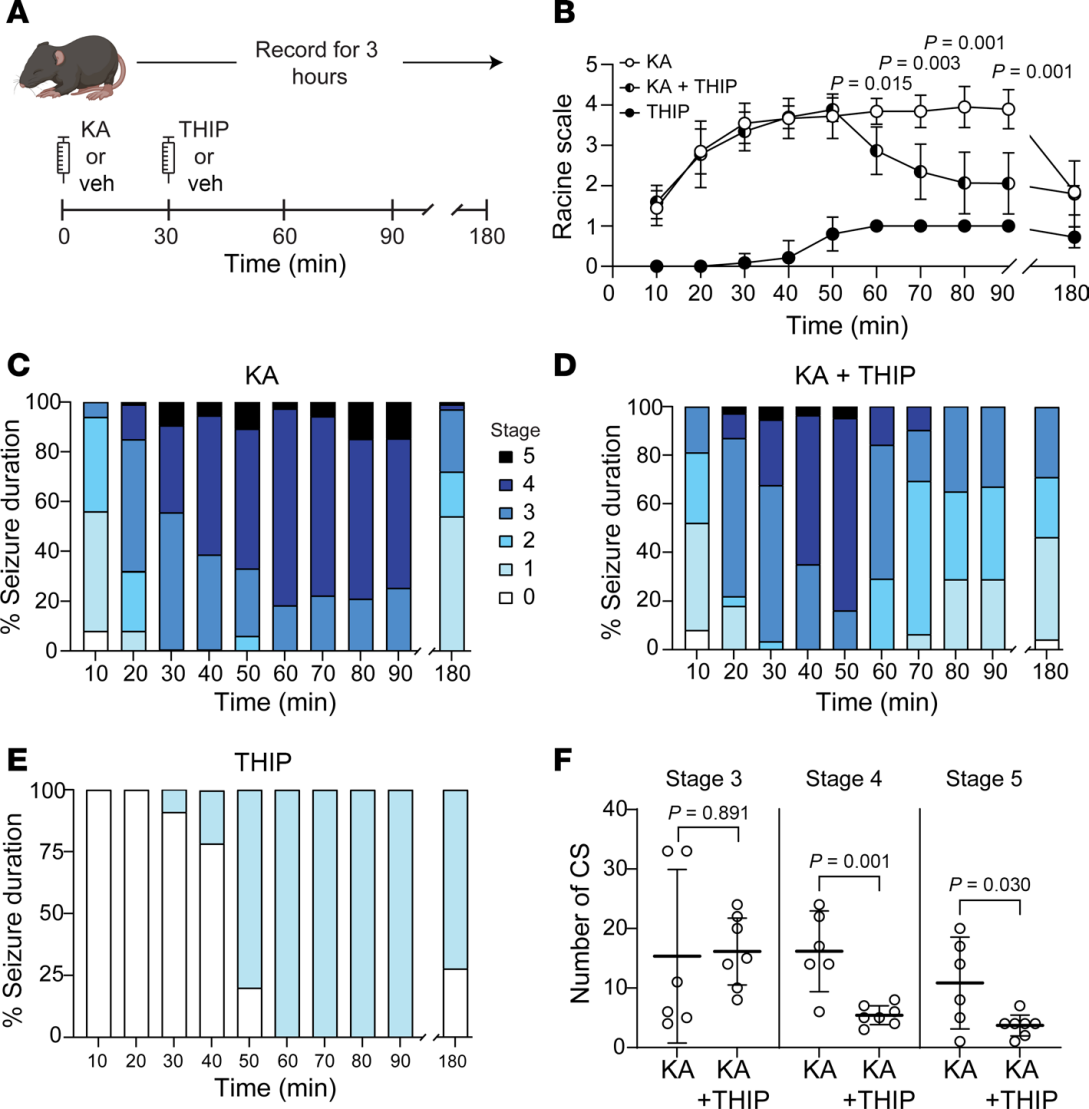
THIP (gaboxadol) reduces behavioral seizure frequency and severity in vivo in neonatal mice. (**A**) In vivo seizure protocol (P8–9). (**B**) Behavioral seizure severity over time (Racine scale) between kainic acid (KA), KA+THIP, and THIP alone over 90 minutes. Two-way ANOVA: time, *F*(3.70, 59.2) = 28.75, *P* < 0.0001; treatment: *F*(2,16) = 167.7, *P* < 0.0001; time × treatment interaction *F*(18, 144) = 12.1, *P* < 0.0001; Tukey’s post hoc test values that are significant between KA vs. KA+THIP are displayed. Comparisons with THIP are not pictured. *n* (mice) = 6 KA, 7 KA+THIP, 6 THIP. (**C**–**E**) Behavioral seizure severity (Racine scale) every 10 minutes in KA, KA+THIP, and THIP. (**F**) Number of convulsive seizures by stage between KA and KA+THIP groups (unpaired *t* test, *n* = same as above per group). Circles indicate an average of mice in **B** and individual mice in **F**. Mean ± 95% CI.

We next assessed the impact of augmenting tonic inhibition with THIP (gaboxadol) on neonatal behavioral seizures in vivo (P8–9). First, we conducted a small pilot study to evaluate the effect of different THIP doses on the behavior of neonatal mice (data not shown), since its in vivo actions have been reported in adults only. We found a notable difference in THIP tolerance between neonatal and adult mice. Prior reports indicate that adult rodents receiving 20–41 mg/kg of THIP survived (37–39), whereas we found that 10 mg/kg was fatal for neonatal mice while 5 mg/kg was highly sedating. Therefore, we chose 3.5 mg/kg to ensure adequate CNS penetration without causing significant sedation. We measured THIP’s effect (3.5 mg/kg s.c.) on behavioral seizures induced by KA (1.5 mg/kg i.p.), a known model that induces status epilepticus and that we have previously used (40, 41). All mice injected with KA had convulsive behavioral seizures (Racine scale 3–5). To enhance the clinical relevance of our study, we administered THIP 30 minutes after KA injection (Figure 8A). The timing of THIP injection was chosen based on the rapid uptake of this drug into the brain after subcutaneous injection, reaching peak concentration within 30 minutes (39). We also included a group that only received THIP. We recorded behavioral events for 3 hours following KA injection and evaluated the first 90 minutes and the last 10 minutes.

After initially behaving similarly, the KA and KA+THIP groups began to diverge significantly in seizure severity at 60 minutes (Figure 8B). Similarly, the duration of the Racine stages differed between groups over time. The KA group primarily exhibited stages 4 and 5 tonic-clonic convulsions, whereas the KA+THIP group displayed stages 1 to 3 between 60 and 90 minutes (Figure 8, C and D). During the final 10 minutes (180 minutes), the groups exhibited similar behavior, with the KA group still showing a few stages 4 and 5 that were absent in the THIP-treated group (Figure 8, B–D). Mice receiving only THIP exhibited ataxia and hypoactivity without complete sedation from the drug, resulting in every mouse having a Racine score of 1 by the 60-minute mark despite not seizing (Figure 8, B and E). No significant difference was observed in the number of stage 3 convulsive seizures with THIP. However, there was a significant reduction in the number of stages 4 and 5 seizures (Figure 8F). These results mirror our ex vivo findings, where enhancing tonic inhibition reduces behavioral seizures.

**Figure 9:**
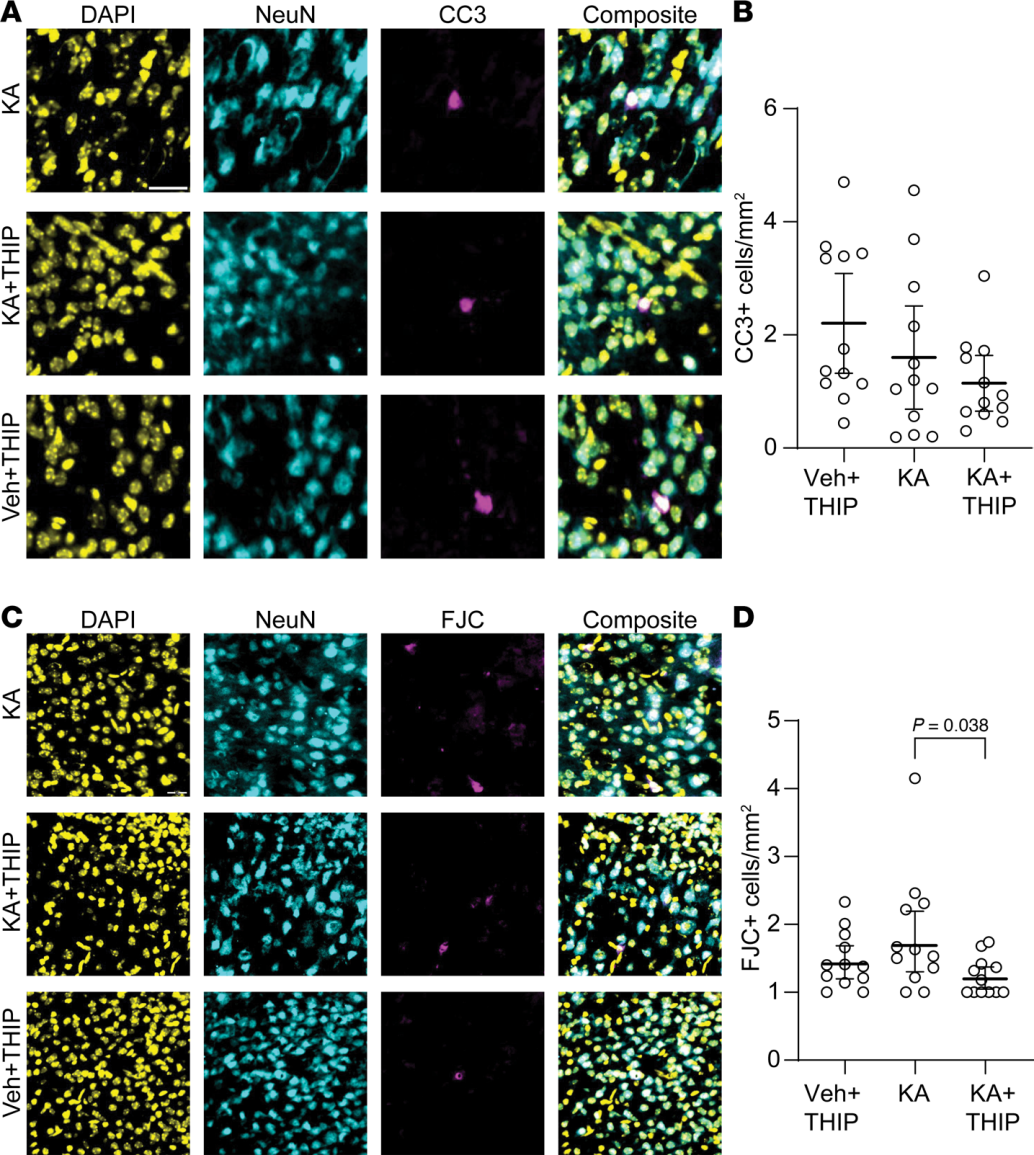
THIP does not cause neuronal apoptosis or degeneration in the neonatal brain in vivo. (**A**) Representative pseudocolored images of the neocortex from each group. Rows: KA, KA+THIP, vehicle plus THIP (Veh+THIP). All images were obtained from mice after 5 hours of behavioral seizures. (**B**) Number of cleaved caspase-3–positive (CC3^+^) neurons after 5 hours of behavioral seizures. One-way ANOVA, *F*(2,33) = 2.22, *P* = 0.124. Mean ± 95% CI. (**C**) Same as **A** but with Fluoro-Jade C (FJC) staining. (**D**) Number of FJC^+^ neurons after 5 hours of behavioral seizures. One-way ANOVA, *F*(2, 33) = 3.69, *P* = 0.036, Tukey’s post hoc test. Data analysis conducted on log-transformed data. *N* = 12 slices in all groups, KA = 6 mice, KA+THIP = 7 mice, Veh+THIP = 6 mice. Circles: individual brain slice. CC3, mean ± 95% CI; FJC, geometric mean ± 95% CI. Scale bar, 25 μm.

Finally, using brain tissue from these mice, we assessed whether THIP administration caused neuronal degeneration or apoptosis (Figure 9). To allow sufficient time for neuronal apoptosis or degeneration to occur, we collected brain tissue 5 hours after the injection of KA (41). We found no statistical difference in neuronal CC3 expression (apoptosis marker) in the neonatal neocortex between the treatment groups (Figure 9, A and B). When we evaluated neurodegeneration using FJC, which was low in all conditions, THIP significantly reduced the number of FJC^+^ neurons in the KA+THIP group compared with the KA group (Figure 9, C and D). Thus, THIP decreased the number of behavioral seizures in the neonatal mice exposed to KA without increasing apoptosis or neurodegeneration.

## Discussion

Our results can be summarized as follows: 1) The α5- and δ-containing GABA_A_R subunits are expressed in the neonatal neocortex, and boosting the δ subunit–containing tonic inhibition reduces acute neonatal SLE while inhibiting the α5 subunit has no effect. 2) Hours of recurrent neonatal SLE, similar to prolonged status epilepticus, increase the expression of α5 and δ subunits in the membrane. 3) This increased expression leads to higher δ-GABA_A_R–mediated tonic currents. 4) Enhancing tonic inhibition is more effective in reducing recurrent neonatal SLE than acute SLE ex vivo. 5) Increasing tonic inhibition decreases KA-induced neonatal behavioral seizures in vivo without enhancing neuronal degeneration or apoptosis.

Our study has some limitations. First, our in vivo THIP dose is lower than our effective dose ex vivo (50 μM), likely ranging between 1.5 μM and 3 μM (31, 39), and it did not eliminate all seizures. We are limited in our ability to use higher doses of THIP in vivo because of sedation-related side effects that can lead to respiratory arrest and the technical challenges associated with intubating neonatal mice. However, human neonates with protracted status epilepticus are typically intubated. Second, our in vivo data were collected without an EEG, and the duration was up to 3 hours, not 8 hours, as in our ex vivo experiments. To create a more clinically relevant model, we would need to perform repeated injections of KA to prolong the in vivo seizures, significantly increasing the risk of mortality at this age. Third, it is unclear whether neuronal apoptosis or neurodegeneration would increase when tonic inhibition is enhanced over more extended periods, as we evaluated it only 5 hours after KA. Further research is needed to evaluate the effects of tonic inhibition modulators on the rates of apoptosis and neurodegeneration across a broader range of subacute and chronic treatments. Finally, much of the above work was conducted in acute brain slices, and as such, must be interpreted with this limitation in mind.

We observed protein expression of the α5- and δ-GABA_A_R subunits in layers IV/V of the neonatal neocortex. Prior work has shown that α5-GABA_A_R mRNA is detectable in the neocortex of the rodent embryos, whereas δ-GABA_A_R mRNA becomes detectable postnatally (21, 22). The expression of the δ subunit has primarily been described in the cerebellum, thalamus, and hippocampal dentate gyrus; however, adult mice also exhibit expression in the cortex (42–44), supporting our current findings. For α5 subunit expression, moderate mRNA expression levels have been observed in layers V/VI of the neocortex and the hippocampus (21, 45, 46). Notably, extrasynaptic α5- and δ-GABA_A_Rs are expressed in both principal cells and interneurons with varying degrees depending on cell type and brain region (47–51). This variability complicates the understanding of how changes in tonic inhibition can lead to alterations in network activity, such as changes in γ oscillations and interneuron excitability (52–54). Therefore, while there is robust expression of the α5 and δ subunits in the neonatal neocortex, further research into cell type–specific expression is needed to elucidate how these differences influence their firing activity and the overall circuit properties.

Inhibiting α5-GABA_A_R–mediated tonic currents with L-655 did not affect neonatal SLE, but THDOC, a δ-containing GABA_A_R neurosteroid, did. It was shown previously that a dose of L-655 specific for α5-GABA_A_R did not alter the dose of pentylenetetrazole required to induce seizures (55), supporting our findings. Interestingly, we found that even when the dose was increased beyond the specificity for α5-GABA_A_Rs, L-655 was not proconvulsant in acute brain slices. It is unclear why this is the case, and we speculate that one possibility is that α5-GABA_A_Rs have a distinct distribution among neuron subclasses.

Several drugs enhance tonic inhibition mediated by δ-containing GABA_A_Rs. Although phenobarbital and pentobarbital can modulate the extrasynaptic δ-containing GABA_A_Rs, this is not their primary mechanism of action (56–59). Although off-target effects of THIP at high doses are likely (60), we did not observe a change in the proportion of α5-mediated tonic conductance. Unfortunately, the δ-knockout mice have compensatory changes in other subunits, including the α4 and γ2 subunits, which makes them less suitable to evaluate this drug (44). Although THIP (gaboxadol) did not progress into clinical trials for status epilepticus, other drugs are being evaluated for the treatment of seizures, including ganaxolone, another drug class that enhances δ-containing GABA_A_R (61–65). For example, in neonatal lambs exposed to perinatal asphyxia, a common cause of neonatal seizures, ganaxolone proved effective in decreasing seizures (64). Similarly, in our experiments, we showed that high doses of THDOC, another neurosteroid, reduced neonatal SLE. The effects of THDOC were similar to those of high doses of THIP. Therefore, even if THIP and THDOC have off-target actions, we demonstrated that both can decrease seizure-like activity.

Our results align with previous research and our earlier findings, demonstrating that enhancing tonic inhibition can lower proximal membrane resistance, shift the resting membrane potential, produce shunting inhibition, and influence the dendritic space constant (16, 31, 66). Specifically, in the newborn brain, despite E_GABA_ being depolarizing at this age, we previously demonstrated that enhancing tonic inhibition reduces neuronal activity by making neurons less excitable (31).

The changes in α5- and δ-GABA_A_R membrane expression during seizures and status epilepticus are complex. In the same time frame during which synaptic GABA_A_Rs are internalized (45 minutes to 2 hours) and benzodiazepines lose clinical efficacy, α5- and δ-GABA_A_Rs either remain in the membrane or increase their expression (28–30). Chronic models of epilepsy demonstrate a significant downregulation of α5- and δ-GABA_A_R membrane expression (67–69); however, there is also evidence of increased δ-GABA_A_R expression (70, 71). Here, we demonstrated that both the α5- and δ-GABA_A_Rs increased their membrane expression after 8 hours of recurrent neonatal SLE, similar to status epilepticus, supporting previous findings in adult models. Most importantly, this increase in membrane expression correlated with a rise in tonic conductance at this age, mainly at the expense of δ-containing GABA_A_Rs. While tonic inhibition increased, there was no change in spontaneous inhibitory postsynaptic current (sIPSC) characteristics besides frequency. The frequency reduction could reflect a decrease in interneuron firing, which has been reported with enhanced tonic inhibition (54).

The most critical observation from our experiments is that tonic inhibition increased after 8 hours of SLE in the neonatal brain ex vivo, which THIP further enhanced. Additionally, the THIP-mediated enhancement was more pronounced at later time points than at earlier ones, and this resulted from increased tonic inhibition after recurrent SLE. Consistent with prior observations, we showed that diazepam did not decrease SLE activity at these delayed time points (24, 72, 73). We observed similar findings in our in vivo experiments, where the duration, severity, and number of behavioral neonatal seizures induced by KA, and ongoing for at least 30 minutes, were reduced after administering THIP. We observed a decrease in ex vivo SLE and in vivo behavioral seizures at 3 hours, but the reduction in the former was not statistically significant. A perfect extrapolation between ex and in vivo conditions is challenging, and in vivo effects involve a more extensive neuronal network compared with the acute brain slice.

Importantly, we found that THIP did not worsen neocortical neuronal apoptosis, as caused by phenobarbital and other common anticonvulsant medications (74, 75), and reduced neuronal degeneration. Similar findings were observed in the CA1 and CA3 regions of neonatal lambs exposed to perinatal asphyxia, where ganaxolone reduced neuronal degeneration (64). Moreover, the lack of increased apoptosis is consistent with what was observed with lacosamide at this age (41). Collectively, our results support the hypothesis that enhancing tonic inhibition is a viable treatment option for neonatal seizures, particularly those that have lasted for hours or are pharmacoresistant, without increasing neurodegeneration or apoptosis.

Intriguingly, patients with mutations that should result in high tonic inhibition paradoxically exhibit epilepsy and neurodevelopmental disorders. For example, children with pathogenic *SLC6A1* mutations that affect the GABA transporter 1 (GAT-1), causing high extracellular GABA levels and increased tonic inhibition in mouse models (76), present with myoclonic-atonic epilepsy and intellectual disability (77, 78). Interestingly, none of the patients so far have a history of neonatal epilepsy. One possible explanation for the incidence of seizures in *SLC6A1* patients is related to the expression of GAT-1, which is mainly found in axons and nerve terminals of GABAergic interneurons (79, 80). Thus, a particular increase in tonic inhibition may lead to a preferential inhibition of interneurons, resulting in increased network excitability. The differential GAT-1 expression might also explain reports of nonconvulsive status epilepticus in patients taking tiagabine, a drug that blocks GABA reuptake (81). Additionally, it has been proposed that neuron-specific GABA transport inhibitors can be proconvulsive (82) compared with blockers of GAT-3, which are primarily astrocytic (83). Finally, chronically elevated extracellular GABA levels could decrease its synaptic release via GABA_B_ receptors (76). A different group of patients, those with *GABRD* (GABA_A_R δ subunit gene) gain-of-function mutations, also exhibit neurodevelopmental disorders and epilepsy, including absence seizures in early childhood (84). Interestingly, high tonic inhibition in thalamocortical neurons is associated with absence epilepsy in rodent models (85). The likelihood of seizures may again depend on the differential expression of the δ subunit in neuronal subtypes and the extracellular GABA concentration level, causing different network outputs (53, 54). Therefore, the role of tonic inhibition in different neuronal subtypes and networks is complex and requires further investigation. Nevertheless, we propose that enhancing tonic inhibition to a level that reduces neuronal firing (31) can help treat neonatal status epilepticus, without affecting long-term network changes caused by chronic treatment.

In conclusion, we demonstrate that, in an ex vivo model, recurrent neonatal seizure-like activity lasting up to 8 hours (akin to status epilepticus) increases tonic inhibition in neocortical pyramidal cells, which is further enhanced by THIP and THDOC, resulting in reduced seizure-like activity. Additionally, THIP decreases neonatal behavioral seizures in vivo without increasing apoptosis or neurodegeneration. Consequently, enhancing tonic inhibition may serve as a treatment for neonatal patients experiencing prolonged or refractory status epilepticus.

## Methods

### Sex as a biological variable.

Sex is a biologically relevant variable, and prior work demonstrates that δ subunit expression fluctuates with the estrus cycle (86, 87). The murine estrus cycle does not begin until P26; therefore, we used both sexes of pups without sexing individual mice.

### Animals.

C57BL/6J mice (P6–9; The Jackson Laboratory, strain 000664) and transgenic P12 Thy1-YFP mice [B6.Cg-Tg(Thy1-YFP)HJrs/J, The Jackson Laboratory, strain 003782] were used for these experiments. The mice were housed in the University of Iowa animal facility. The Institutional Animal Care and Use Committee of the University of Iowa approved the experiments.

### Acute brain slices.

Mice were anesthetized with isoflurane and decapitated per approved protocol. The brain was placed in ice-cold aCSF containing NaCl (120 mM), KCl (3.3 mM), CaCl_2_ (1.3 mM), MgCl_2_ (2 mM), NaH_2_PO_4_ (1.25 mM), NaHCO_3_ (25 mM), and d-glucose (10 mM) with a pH of 7.3–7.4 when bubbled with 95% O_2_ and 5% CO_2_. Coronal brain slices, 450 μm thick, were cut using a vibratome (Camden Instruments 7000smz-2) while submerged in aCSF containing 2 mM kynurenic acid and 2 mM MgCl_2_ to block glutamate receptors. We and others have used this thickness to record seizure-like activity in the past (7, 31, 34). Furthermore, we have not observed an increase in propidium iodide (cell death marker) staining in slices of this thickness (88). Brain slices were placed in a submersion holding chamber containing aCSF (1.3 mM MgCl_2_) at room temperature (RT) for 30 minutes. Next, the temperature was gradually increased and maintained at 30°C until the time of the experiment. Slices were stored at 30°C for at least 1 hour before experimental use.

### Recurrent SLE.

Neonatal coronal brain slices containing the motor and somatosensory regions of C57BL/6J mice were placed in a custom-made submersion chamber containing either regular aCSF (control) or Low-Mg^2+^ aCSF (SLE) for 8 hours. For the recurrent seizure time course experiments, slices were removed from the Low-Mg^2+^ aCSF incubation at different time points between 0 and 8 hours.

### Field electrophysiology.

Brain slices were placed in an interface recording chamber and perfused with Low-Mg^2+^ (0 mM MgCl_2_) aCSF to induce SLE. The slices were maintained at 32°C–34°C and oxygenated with 95% O_2_ and 5% CO_2_. ACSF-filled glass electrodes were placed in the neocortex (motor and somatosensory regions, up to the anterior hippocampus, layers IV/V) or the hippocampal CA1 stratum pyramidale. A stereomicroscope (AmScope) was used to visualize electrode placement. Extracellular field potentials were recorded using a low-noise differential amplifier (100× gain, DP-311, Warner Instruments) and digitized at 2 kHz with an analog-to-digital converter (IX/408, iWorx Systems Incorporated). An FFT was used to measure the power of SLE during the baseline and subsequent perfusion of the different compounds through a custom-written macro programmed in Igor Pro v9 (WaveMetrics) as previously described (7). SLE characteristics were analyzed using another Igor Pro custom-written macro (89), applying identical time frames (900 seconds) for baseline and drug conditions. The parameters for SLE analysis were event threshold of 3× baseline, minimum event length 0.04 seconds, and dead time 1 second. Events lasting ≥10 seconds were classified as ictal, while events lasting <10 seconds were classified as interictal (90).

### Whole-cell patch-clamp recordings.

Brain slices were transferred to a submerged chamber and perfused with aCSF (32–34°C; 95% O_2_, 5% CO_2_) containing kynurenic acid (2 mM) to block glutamatergic transmission. Pyramidal cells in the neocortex (somatosensory and motor, layer IV/V) were identified by IR-Dodt-Gradient Contrast video microscopy (Olympus BX51WIF, 40× water immersion objective, with an infracontrast Dodt-Gradient-Contrast, Luigs & Neumann), imaged with a CS505MU CMOS camera (Thorlabs), and recorded with an integrated patch-clamp amplifier (Double-IPA, Sutter Instruments). For tonic and phasic inhibitory current measurements, micropipettes (2–5 MΩ) contained the following internal solution: CsCl (140 mM), MgCl_2_ (1 mM), HEPES (10 mM), EGTA (0.15 mM), NaCl (4 mM), Mg-ATP (2 mM), Na_2_-GTP (0.3 mM), and QX-314 (5 mM), pH ~7.25, ~280 mOsm. Whole-cell voltage-clamp recordings were performed at a holding potential of –70 mV, acquired at 10 kHz, and low-pass–filtered at 3 kHz. Series resistance and whole-cell capacitance were estimated and compensated, and the recordings were discontinued if the series resistance increased by more than 25% during the experiment. Slices were perfused with aCSF or the different drugs to establish a stable baseline. After this, bicuculline methiodide (10 mM, 20 μL) was injected into the perfusion chamber to block all GABA_A_Rs.

### In vivo seizures.

Seizures were induced with KA (1.5 mg/kg i.p.) in neonatal mice (P8–9), following a protocol as described in our prior publication (41). Experimental groups received gaboxadol (THIP; 3.5 mg/kg subcutaneously) 30 minutes after KA injection, with saline used as a vehicle for controls. Mice were monitored for behavioral seizures and staged based on the modified Racine scale: Stage 0: normal; Stage 1: hypoactivity, immobilization, or absence-like immobility, and freezing; Stage 2: head nodding, hunched back posture, masticatory movements, and facial or manual automatisms; Stage 3: myoclonus (righting reflex is preserved), myoclonic jerks, Straub tail, rearing, and forelimb clonus; Stage 4: rearing, tonic seizure, and falling on its side; Stage 5: tonic-clonic seizure with loss of righting reflex, falling on its back, wild running and jumping (91). For back-to-back events of the same seizure stage, we considered them separate if there was a ≥30-second gap between them. All seizures were video-recorded for 3 hours at 20 frames per second. The first 90 minutes and final 10 minutes were staged (from 0 to 5) with the reviewer masked to the experimental conditions. Mice were observed for 2 additional hours and then euthanized at the end of the experiment (5 hours total). Brains were extracted and fixed with 4% paraformaldehyde in phosphate-buffered saline for immunofluorescence. The brains were then cryopreserved in 30% sucrose until saturated, embedded in gelatin, flash-frozen in liquid nitrogen, and processed into 10 μm sections using a cryostat (CM3050S, Leica). Brains were sliced at the prehippocampal neocortex to eliminate variability between brain regions, and we analyzed at least 1 slice from every mouse in each treatment group.

### Tonic inhibition analysis.

The tonic inhibitory conductances were calculated using a custom-written IgorPro macro (92). The tonic current was obtained by fitting a Gaussian to the all-points histogram over a specific epoch (60 seconds for each condition). The Gaussian was fitted to the distribution portion not skewed by synaptic events during the baseline condition. We determined the magnitude of the net tonic current by subtracting the current recorded in the presence of bicuculline from the previous drug conditions. The tonic currents were expressed as conductances and normalized to the membrane capacitance (pS/pF) to account for cell size variability. Electrophysiological recordings were analyzed after acquisition using SutterPatch, IgorPro, and GraphPad Prism.

### SIPSC analysis.

sIPSCs were detected and analyzed using SutterPatch (Sutter Instruments). The synaptic event template was rise = 1.5 ms, decay = 15 ms, and 4 pA threshold amplitude. The detected events were verified manually. sIPSCs were analyzed during the baseline segment analysis window during whole-cell recordings.

### Immunofluorescence.

For labeling of GABA_A_R subunits, slide-mounted C57BL/6J brain sections (10 μm, P8) or Thy1-YFP brain sections (30 μm, P12) were labeled with the α5 or δ GABA_A_R subunits with a primary antibody via the following steps: 1) Sections were permeabilized with 0.2% Triton X-100 for 30 minutes at RT. 2) Sections were incubated with α5 (1:400, EMD Millipore AB9678) or δ (1:200, Alomone Labs AGA-014) primary antibody overnight at 4°C, then with a secondary antibody (α5 1:800, δ 1:400; Invitrogen A32740 goat anti-rabbit IgG, Alexa Fluor 594). Brain sections were additionally labeled with NeuN (1:200, Anti-NeuN Antibody conjugate, clone A60, Alexa Fluor 555, Sigma-Aldrich). Sections were coverslipped with Vector Laboratories VECTASHIELD Vibrance Antifade Mounting Medium and imaged on a Leica DFC 7000 T confocal microscope. For labeling apoptosis, slide-mounted brain sections were labeled with CC3 following these steps: 1) Sections were permeabilized with 0.2% Triton X-100 for 30 minutes at RT. 2) Sections were incubated with CC3 primary (1:100, Cell Signaling Technology D175 catalog 5A1E) overnight at 4°C, followed by a secondary antibody (1:250, Invitrogen A27034 goat anti-rabbit IgG, Alexa Fluor 488) and NeuN Conjugate (1:200, Sigma-Aldrich MAB377A5, Alexa Fluor 555) for 2 hours at RT. 3) Sections were coverslipped with VECTASHIELD Vibrance Antifade Mounting Medium with DAPI and imaged using a Leica DFC 7000 T confocal microscope. For FJC staining, we followed modified steps from the Biosensis Ready-to-Dilute Fluoro-Jade C Staining Kit after secondary incubation with the NeuN conjugate: 1) Sections were pretreated with potassium permanganate (9.5 parts H_2_O, 0.5 parts potassium permanganate) for 10 minutes at RT and then rinsed with deionized water. 2) Sections were treated with FJC stain (7 parts H_2_O, 2 parts FJC, 1 part DAPI) for 20 minutes at RT and then rinsed with deionized water. 3) Sections were rapidly dried in a Boekel Scientific RapidFISH Slide Hybridizer and then cleared in xylene for 2 minutes at RT. 4) Once dried, sections were coverslipped with Vector Laboratories Vibrance Antifade Mounting Medium (no DAPI).

### Imaging and reconstruction.

Brain slides were imaged under a 60× objective on a Leica DFC 7000T confocal microscope with the following filters: green: excitation (ex) 480/40, emission (em) 527/30, dichroic (dc) 505; red: ex560/40, em645/75, dc595; blue: ex360/40, em470/40, dc400. Green was used to image Thy1-YFP, FJC, or CC3; blue to image DAPI; and red for imaging the α5 or δ subunits or NeuN. Using the Leica Application Suite X program, simultaneous *Z*-stacks and tile scans were performed to create 3-dimensional images. Each image from an experimental group was recorded at 12 bits (2.20 pixels/μm) with identical fluorophore exposure times and gain settings for all slides. Image processing was performed in Imaris 3D (Oxford Instruments) to generate surfaces from each channel, which were overlaid. Imaging analysis (ImageJ, NIH) was performed in the neocortex in a blinded manner with respect to treatment. To analyze CC3, a manual threshold was used to obtain cell boundaries and generate regions of interest (ROIs) in the CC3 channel (size: 15–150 μm^2^; circularity: 0.3–1.00). CC3 ROIs were overlaid onto the DAPI channel to verify that each ROI corresponded to a cell. CC3 ROIs were then overlaid onto the NeuN channel, where cells were considered double-labeled for CC3 and NeuN if the NeuN signal within the CC3 ROI was 2.5 times higher than the whole-slice NeuN or DAPI channel mode. For analysis of FJC imaging, we used 2.5 times the whole-slice mode to obtain cell boundaries and generate ROIs in the FJC channel (size: 15–150 μm^2^; circularity: 0.3–1.00). FJC ROIs were then overlaid onto the DAPI channel, where cells were considered to have valid FJC expression if the DAPI signal within the FJC ROI was 2.5 times higher than the whole-slice DAPI channel mode.

### Tissue fractionation.

To analyze membrane expression of the α5 and δ GABA_A_R subunits, the neocortex was dissected from acute neonatal brain slices, which had been incubated in aCSF or Low-Mg^2+^ aCSF (8 hours). Dissected neocortices were placed into conical tubes containing cytoplasmic extraction buffer with added HALT protease (1:100) using the Thermo Fisher Scientific Subcellular Protein Fractionation Kit for Tissues (catalog 87790). The tissue was hand-homogenized (10 times) with a conical tube pestle, and 1/5 of the lysate was removed, sonicated, and labeled “total lysate.” The remaining lysate was centrifuged at 500*g* for 5 minutes at 4°C, and the supernatant was removed and labeled “cytoplasmic lysate.” Membrane extraction buffer with HALT protease (1:100) was added to the pellet, which was vortexed, reconstituted, and incubated with light mixing for 30 minutes at 4°C. The sample was then centrifuged at 3,000*g* for 5 minutes at 4°C, and the supernatant was removed and labeled “membrane lysate.”

### Western blot.

To visualize each receptor subunit, 80 μg of protein was loaded per well and separated on a NuPage 4%–12% Bis-Tris Gel (1.5 mm × 10 well, Invitrogen, 58005). Control gels for Na^+^/K^+^ ATPase were loaded with 20 μg or 5 μg. The gel was transferred to an Immobilon-P 0.45 μm PVDF (Merck Millipore, IPVH00010) membrane and was placed in a blocking solution (5% nonfat milk) for 1 hour. Afterward, the membrane was transferred to a blocking solution containing the appropriate primary antibody (1:1,000) overnight at 4°C. The following day, the membranes were rinsed 3 times with PBS and placed in a blocking solution containing the secondary antibody (HRP 1:2,000, Bio-Rad, 1706515) for 1 hour. Membranes were then rinsed with TBS with Tween 3 times, and immunoreactivity was detected with SuperSignal West Pico PLUS Chemiluminescent Substrate (Thermo Fisher Scientific, 34577) via an iBright FL1500 imaging system (Invitrogen, A44241). Bands were quantified within the linear exposure range using ImageJ, and band intensity was normalized to amido black total protein stain. To perform the amido black total protein stain, the blots were first washed with Milli-Q water (MilliporeSigma) after imaging. Blots were then incubated in amido black solution for 2–3 minutes and rinsed in Milli-Q water. Stained blots were dried and then imaged. The total protein quantification for each lane was performed using ImageJ.

### Reagents.

THIP, bicuculline methiodide, kynurenic acid (4-hydroxyquinoline-2-carboxylic acid), diazepam, α5-GABA_A_R primary (AB9678), and THDOC were obtained from Sigma-Aldrich. QX-314 was obtained from Tocris Bioscience. The δ-GABA_A_R primary was acquired from Phosphosolutions. L-655, KA, and the secondary antibodies were obtained from Thermo Fisher Scientific. All drugs (except L-655 and THDOC) were prepared in deionized water and diluted to the appropriate concentrations in aCSF. L-655 and THDOC stocks were prepared in DMSO and diluted to a final maximal concentration of 0.1% DMSO. Diazepam was prepared as stock in 100% ethanol and diluted to 2 μM with a final ethanol concentration of 0.04%.

### Statistics.

The normality of distributions was evaluated using the Shapiro-Wilk test and QQ plots. Gaussian-distributed data are presented as mean ± 95% CI while non-Gaussian data are presented as median ± IQR. A 1-sample 2-tailed *t* test was used for single-sample comparisons. Comparisons between 2 paired groups were performed using the paired 2-tailed *t* test (parametric) or the Wilcoxon test (nonparametric), while comparing of 2 unpaired groups was performed using the unpaired 2-tailed *t* test (parametric). One-way ANOVA was used to compare multiple parametric data groups, followed by Tukey’s post hoc test or Šídák’s multiple-comparison test. For FJC analysis, some slices had 0 counts, so all counts were transformed using Y = log(Y+1) to obtain a normal distribution. For in vivo seizure analysis, the severity was averaged every 10-minute epoch over the 90-minute analysis period, and the average was analyzed using a 2-way repeated measures ANOVA with treatment, time, and treatment × time as sources of variation. When a significant interaction was observed, the mean severity scores were compared between each group using a Tukey post hoc test. Statistical significance was set as *P* < 0.05. We used IgorPro v9 and Prism 9 (GraphPad Software) for data analysis.

### Study approval.

All experiments were conducted in accordance with a protocol approved by the University of Iowa Institutional Review Board.

### Data availability.

Values underlying all graphed data are in the Supporting Data Values file, and any additional relevant data will be available directly from the corresponding author.

## Author contributions

GTL and JG designed the research. GTL performed the experiments and analyzed the data. GFB advised on in vivo seizure recordings and edited the manuscript. MLS advised on molecular biology techniques and edited the manuscript. GTL wrote the first draft, and JG provided guidance and editing of the manuscript.

## Funding support

This work is the result of NIH funding, in whole or in part, and is subject to the NIH Public Access Policy. Through acceptance of this federal funding, the NIH has been given a right to make the work publicly available in PubMed Central.

- JG by NIH/National Institute of Neurological Disorders and Stroke R01NS115800 and the Iowa Neuroscience Institute.
- The University of Iowa Hawkeye Intellectual and Developmental Disabilities Research Center P50 HD10355.
- The University of Iowa Interdisciplinary Graduate Program in Neuroscience’s training grant (NS007421).
- The University of Iowa Medical Scientist Training Program’s training grant (5T32GM139776-04).

## Supplementary Material

Unedited blot and gel images

Supporting data values

## Version 1 09/16/2025

In-Press Preview

## Version 2 11/10/2025

Electronic publication
