# Supplementary figures and images for: Recurrent neonatal seizures increase tonic inhibition and respond to enhancers of δ-containing GABAA receptors
Source: JCI Insight. 2025 Sep 16;10(21):e196152. doi: 10.1172/jci.insight.196152 (PMC12643521; doi:10.1172/jci.insight.196152)

Full unedited gel for Figure 4 (5).pdf  
940 x 864

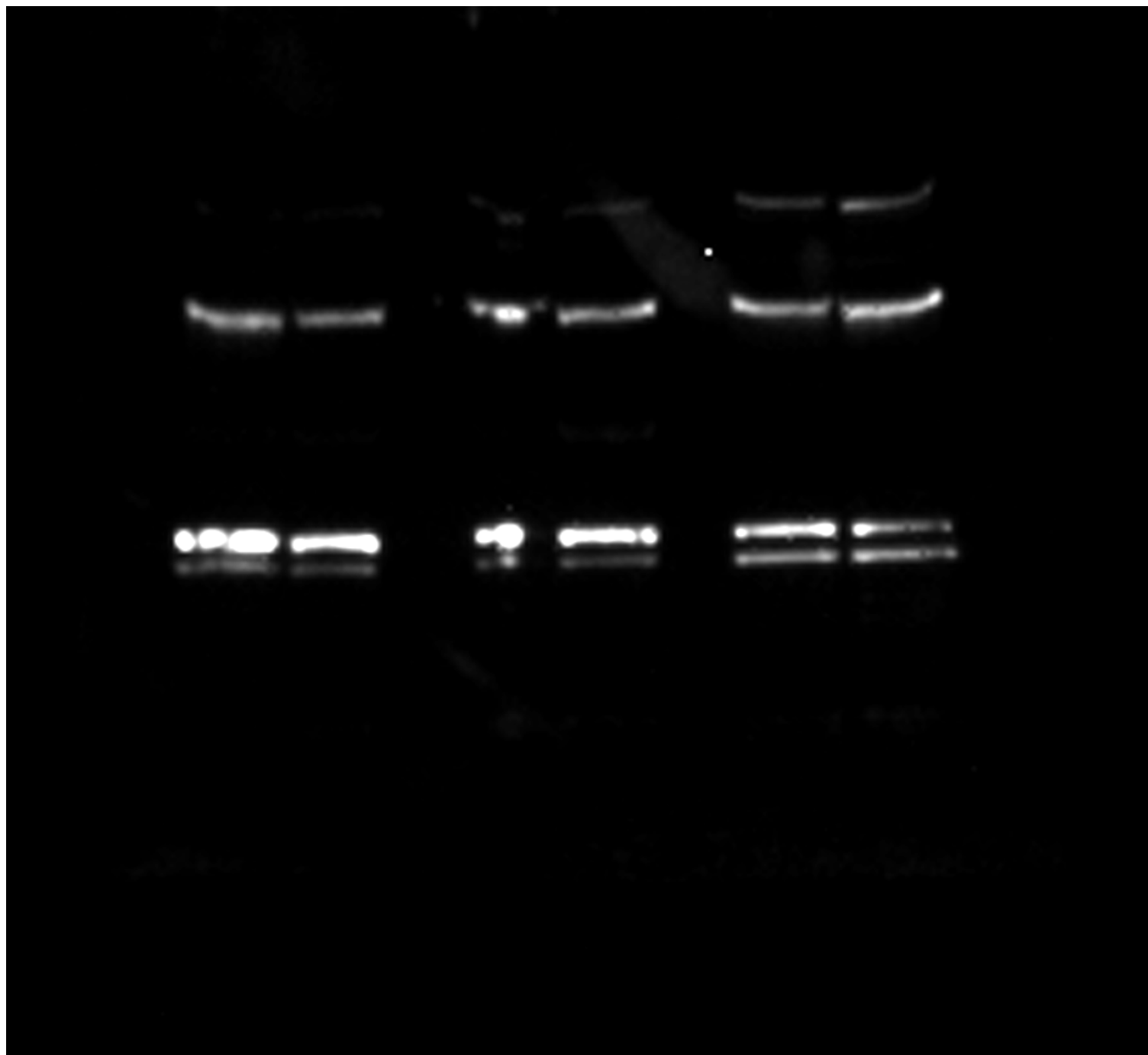

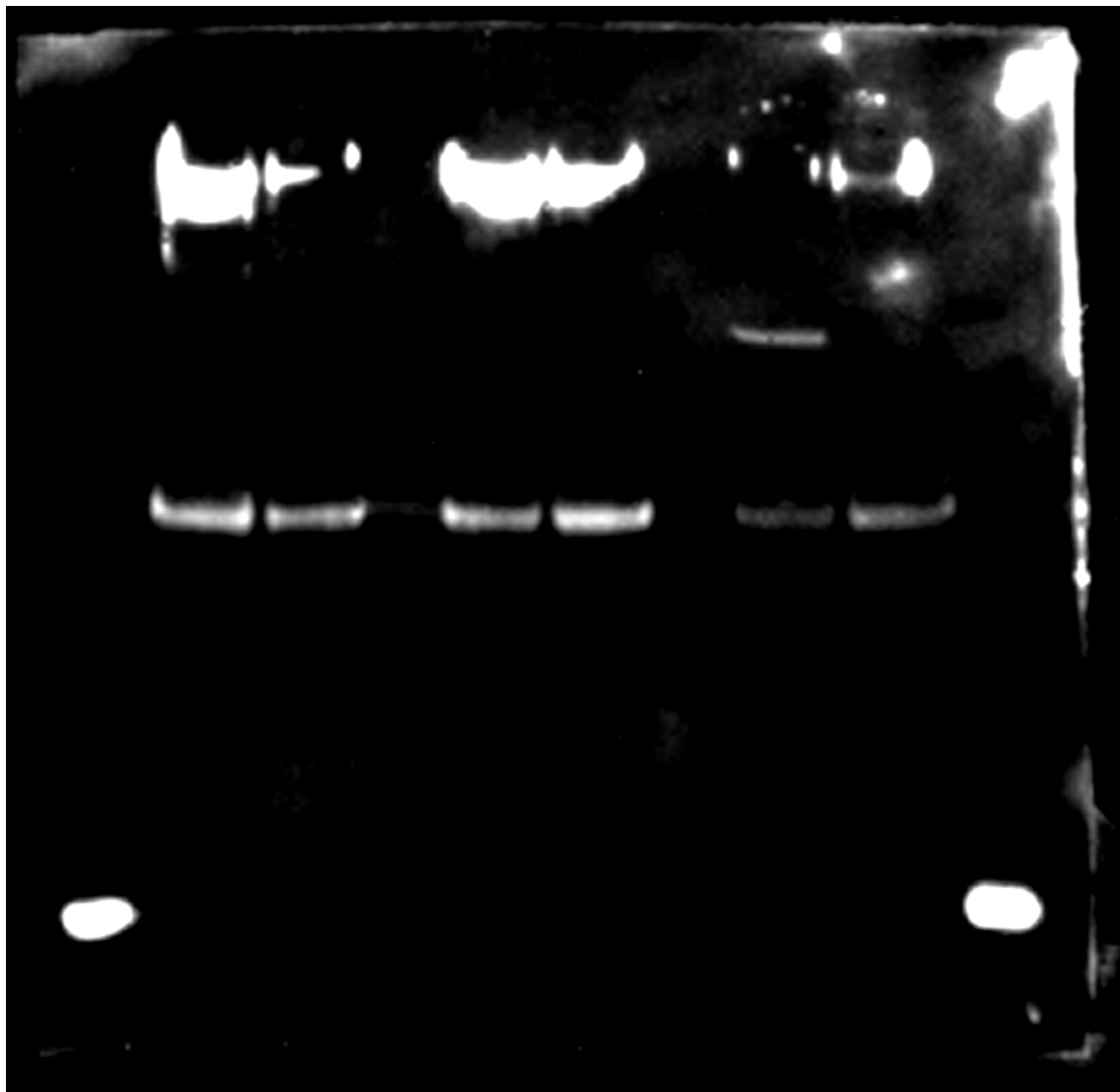

Supplement: Unedited blot and gel images [file jciinsight-10-196152-s030.pdf]
